# PASylated Thymosin α1: A Long-Acting Immunostimulatory Peptide for Applications in Oncology and Virology

**DOI:** 10.3390/ijms22010124

**Published:** 2020-12-24

**Authors:** Uli Binder, Arne Skerra

**Affiliations:** 1 XL-protein GmbH, Lise-Meitner-Str. 30, 85354 Freising, Germany; 2 Lehrstuhl für Biologische Chemie, Technische Universität München, Emil-Erlenmeyer-Forum 5, 85354 Freising, Germany

**Keywords:** biobetter, cancer, COVID-19, drug delivery, half-life extension, immunostimulatory peptide, PASylation, pharmacokinetics, thymosin alpha 1, viral disease

## Abstract

Thymosin α1 (Tα1) is an immunostimulatory peptide for the treatment of hepatitis B virus (HBV) and hepatitis C virus (HCV) infections and used as an immune enhancer, which also offers prospects in the context of COVID-19 infections and cancer. Manufacturing of this N-terminally acetylated 28-residue peptide is demanding, and its short plasma half-life limits in vivo efficacy and requires frequent dosing. Here, we combined the PASylation technology with enzymatic in situ N-acetylation by RimJ to produce a long-acting version of Tα1 in *Escherichia coli* at high yield. ESI-MS analysis of the purified fusion protein indicated the expected composition without any signs of proteolysis. SEC analysis revealed a 10-fold expanded hydrodynamic volume resulting from the fusion with a conformationally disordered Pro/Ala/Ser (PAS) polypeptide of 600 residues. This size effect led to a plasma half-life in rats extended by more than a factor 8 compared to the original synthetic peptide due to retarded kidney filtration. Our study provides the basis for therapeutic development of a next generation thymosin α1 with prolonged circulation. Generally, the strategy of producing an N-terminally protected PASylated peptide solves three major problems of peptide drugs: (i) instability in the expression host, (ii) rapid degradation by serum exopeptidases, and (iii) low bioactivity because of fast renal clearance.

## 1. Introduction

Thymosin α1 (Tα1) is an immunostimulatory peptide initially isolated from calf thymus [1] and also abundant in humans. Tα1 is synthesized as the N-terminal moiety of the highly acidic (pI = 3.7) prothymosin α (ProTα), a peculiar cytoplasmic protein due to the absence of any sulfur-containing as well as aromatic side chains and, in particular, its random coil structure under physiological conditions [2,3]. Cleaved by the lysosomal asparaginyl endopeptidase (legumain; δ-secretase), the N-terminally acetylated 28-residue peptide Tα1 gets released [4]. Tα1 plays a significant role in activating and regulating various cells of the immune system [5], e.g., by stimulating toll-like receptor (TLR)-2 and TLR-9 on myeloid and plasmacytoid dendritic cells (DCs), which results in the secretion of immune-related cytokines [6,7]. Furthermore, Tα1 increases the number of activated T helper (Th) cells and provokes a shift towards the Th1 subclass, thus promoting the cell-mediated immune response. In addition, Tα1 was reported to reduce apoptosis of immune cells [8] and to upregulate the expression of major histocompatibility complex I (MHC I) molecules [9] as well as tumor antigens [10]. Interestingly, Tα1 mediates increased intracellular glutathione (GSH) levels [11], which not only inhibits the growth of certain cancer cells in vitro [7,12], but also blocks the assembly of virus particles by hindering disulfide bond formation that is required for envelope glycoprotein oligomerization [13]. These features offer a broad range of clinical applications for Tα1 [14].

In fact, a chemically synthesized Tα1 peptide drug has been marketed for more than 20 years under the trade name Zadaxin™ in more than 30 countries. Zadaxin™ is clinically approved for the treatment of chronic hepatitis B (HBV), chronic hepatitis C (HCV), as a vaccine adjuvant, and as adjuvant therapy for chemotherapy-induced immune suppression [7]. Of note, the use of Tα1 has recently undergone reassessment in the context of modern cancer immunotherapies, as there is indication that this peptide may support immune checkpoint inhibition. Preclinical data provide evidence that Tα1 transforms so called cold tumors, which poorly respond to immune checkpoint inhibitors, into highly lymphocyte-infiltrated tumors and, thus, boosts therapeutic efficacy [15]. Indeed, a retrospective study evaluating clinical phase II data reported an increase in overall survival of melanoma patients receiving sequentially Tα1 and the immune checkpoint inhibitor ipilimumab, a CTLA-4-blocking antibody, compared to ipilimumab therapy alone [16]. Moreover, Tα1 was shown to prevent intestinal toxicity in a murine model of anti-CTLA-4-induced colitis, hence contributing to an improved safety profile of immune checkpoint inhibitors [17]. Beyond that, emerging indications such as cystic fibrosis [18], multiple sclerosis [19], and sepsis [20] further emphasize the huge potential of this small therapeutic peptide. Finally, with the outbreak of the COVID-19 pandemic, Tα1 recently gained new attention in virology. Clinical symptoms of COVID-19 to some extent resemble a pathogen-induced sepsis, which justifies immunomodulatory Tα1 treatment [21]. Along these lines, Chinese medical staff members at high risk of COVID-19 infection already received a weekly Tα1 injection in combination with human interferon 1 (hIFN-α) nasal drops as a prophylactic measure [22]. Furthermore, a recent retrospective study in treated Chinese patients revealed that Tα1 significantly reduced the mortality of severe COVID-19 by restoration of lymphocytopenia and reversion of exhausted T cells [23].

However, there are two major drawbacks of the currently marketed Tα1, both of which are related to its peptidic nature. First, the biopharmaceutical production of Tα1 is challenging. Apart from the posttranslational modification via N-terminal acetylation, chemical synthesis of Tα1 is complex, and obstacles comprise the large number of required protecting groups as well as the aggregation tendency of intermediates during synthesis [24]. Furthermore, the overall yield of the solid-phase synthesis is low, typically reaching only around 25% [25]. On the other hand, the biotechnological production as a recombinant peptide in an economic manner has failed so far. Short peptides, in general, are quickly degraded in the bacterial cytoplasm; thus, efficient one-step production of mature Tα1 in *E. coli* is not feasible. An alternative would be expression as part of a larger protein such as natural ProTα [26], as artificial concatamers [27], in fusion with an intein [28] or in fusion with a highly expressed bacterial protein like thioredoxin [29]. In each case, enzymatic or chemical cleavage is necessary to liberate the mature peptide, which complicates the biopharmaceutical downstream process. Second, after administration in vivo, the small peptide Tα1 is quickly eliminated via renal filtration with a terminal plasma half-life in humans of less than 3 h [30]. This limits its clinical efficacy and, to maintain viable drug levels, would require twice daily dosing.

In the present study, we applied the PASylation^®^ technology in order to overcome both of these obstacles of the currently available Tα1 drug and to create a long-lasting N-terminally acetylated therapeutic peptide, also offering cheap and efficient biotechnological production in *E. coli*. To this end, we combined fusion with a 600-residue polypeptide comprising the small natural L-amino acids Pro, Ala, and Ser [31] with in situ N-acetylation by overexpressing the host cell N-acetyltransferase RimJ [32]. The genetically encoded uncharged “PAS” sequence is highly soluble and structurally disordered, with an expanded hydrodynamic volume, thus showing a biophysical behavior very similar to the chemical polymer polyethylene glycol (PEG), which has been utilized for plasma half-life extension of a series of other therapeutic peptides and proteins [33,34,35].

## 2. Results

### 2.1. Cloning and Bacterial Production of PASylated Tα1

**Figure 1:**
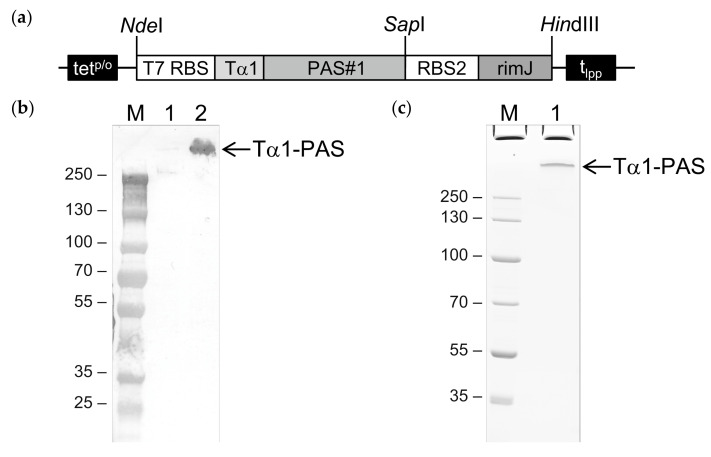
Bacterial production of N-terminally acetylated Tα1-PAS. (**a**) Expression vector for Tα1-PAS and the N-acetyltransferase RimJ from a bicistronic operon under control of the inducible tet^p/o^. The gene cassette cloned on pASK75 comprises the T7 phage ribosome binding site (T7 RBS) followed by the coding sequence for human Tα1 fused to a 601-residue PAS#1 sequence, the second RBS, the coding sequence for RimJ, and the lipoprotein terminator (t_lpp_). (**b**) Western blot analysis after 4–12% SDS–PAGE of Tα1-PAS in the *E. coli* whole cell lysate using an antibody directed against the PAS#1 sequence. M, protein size standard; lane 1, whole cell extract before induction; lane 2, whole cell extract 15 h after induction. (**c**) 10% SDS-PAGE of purified Tα1-PAS. M, protein size standard; lane 1, Tα1-PAS after ammonium sulfate precipitation, subtractive anion exchange (AEX), cation exchange (CEX), and final AEX chromatography.

To achieve C-terminal PASylation of N-terminally acetylated Tα1, a plasmid harboring a bicistronic operon was constructed to allow the simultaneous expression of human Tα1 (UniProtKB ID: P06454; residues 2–29), C-terminally fused with a PAS polypeptide comprising 601 amino acids [34], and the *E. coli* N-acetyltransferase RimJ (UniProtKB ID: P0A948) based on the vector pASK75 (Figure 1) [36]. In a parallel attempt, a plasmid encoding an N-terminally PASylated Tα1 was constructed, again, using plasmid pASK75 as the backbone (this time omitting the RimJ cistron, see below). Cytoplasmic gene expression was performed in both cases on a 2 L shake flask scale using the *E. coli* strain NEBexpress under control of the chemically inducible tet promoter/operator [36].

The whole cell lysate was analyzed prior to and 15 h after induction by Western blotting. Using a monoclonal antibody that recognizes an epitope of the PAS#1 sequence, a distinct band with an approximate molecular size above 250 kDa was detected (Figure 1b), which demonstrated successful bacterial expression of the full-length C-terminally PASylated Tα1 (Tα1-PAS) without signs of degradation. Of note, the unusually slow migration of Tα1-PAS (52.7 kD) in sodium dodecyl sulfate polyacrylamide gel electrophoresis (SDS-PAGE) is well known for PASylated proteins [31,37] and can be explained by the poor binding of SDS (which provides the electrophoretic driving force) to the strongly hydrophilic PAS sequence.

### 2.2. Purification and In Vitro Characterization of PASylated Tα1

The uncharged PAS moiety, which does not alter the isoelectric point of the target peptide, facilitates classical protein precipitation by ammonium sulfate, thus providing an efficient and inexpensive purification step. After adjusting the cleared whole cell extract prepared by mechanical cell lysis to 30% ammonium sulfate saturation, most of the host cell proteins remained in solution while both PAS-Tα1 and PAS-Tα1 were selectively recovered as a precipitate. To remove residual *E. coli* proteins, the redissolved precipitate was subjected to ion exchange chromatography on a salt-tolerant anion exchange (AEX) resin at pH 8.5. Even though the PASylated Tα1 peptide with a calculated pI of 4.3 [38] for both versions should be negatively charged under these conditions and, hence, is expected to adsorb to the resin, the recombinant fusion proteins were quantitatively found in the flow-through. Possibly, the voluminous PAS polymer partially shields the small peptide from ionic interactions with the chromatography matrix. Nevertheless, this step resulted in efficient depletion both of residual host cell proteins and of bacterial endotoxins.

**Figure 2:**
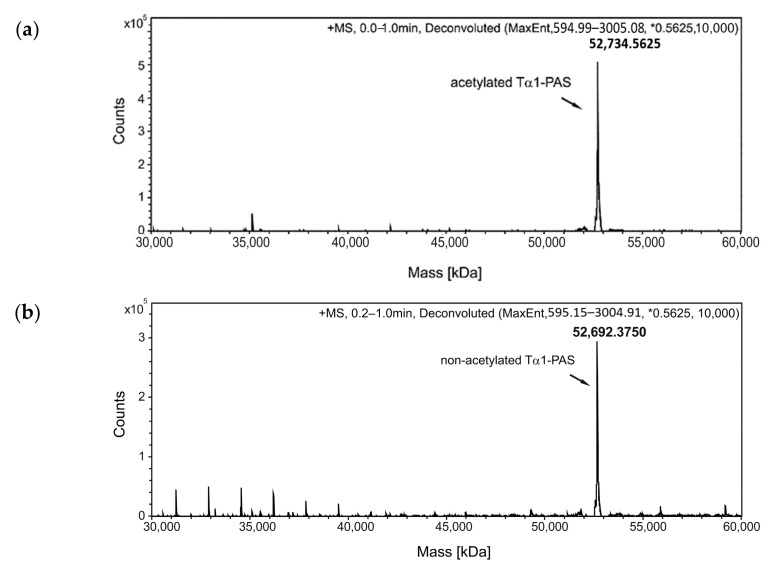

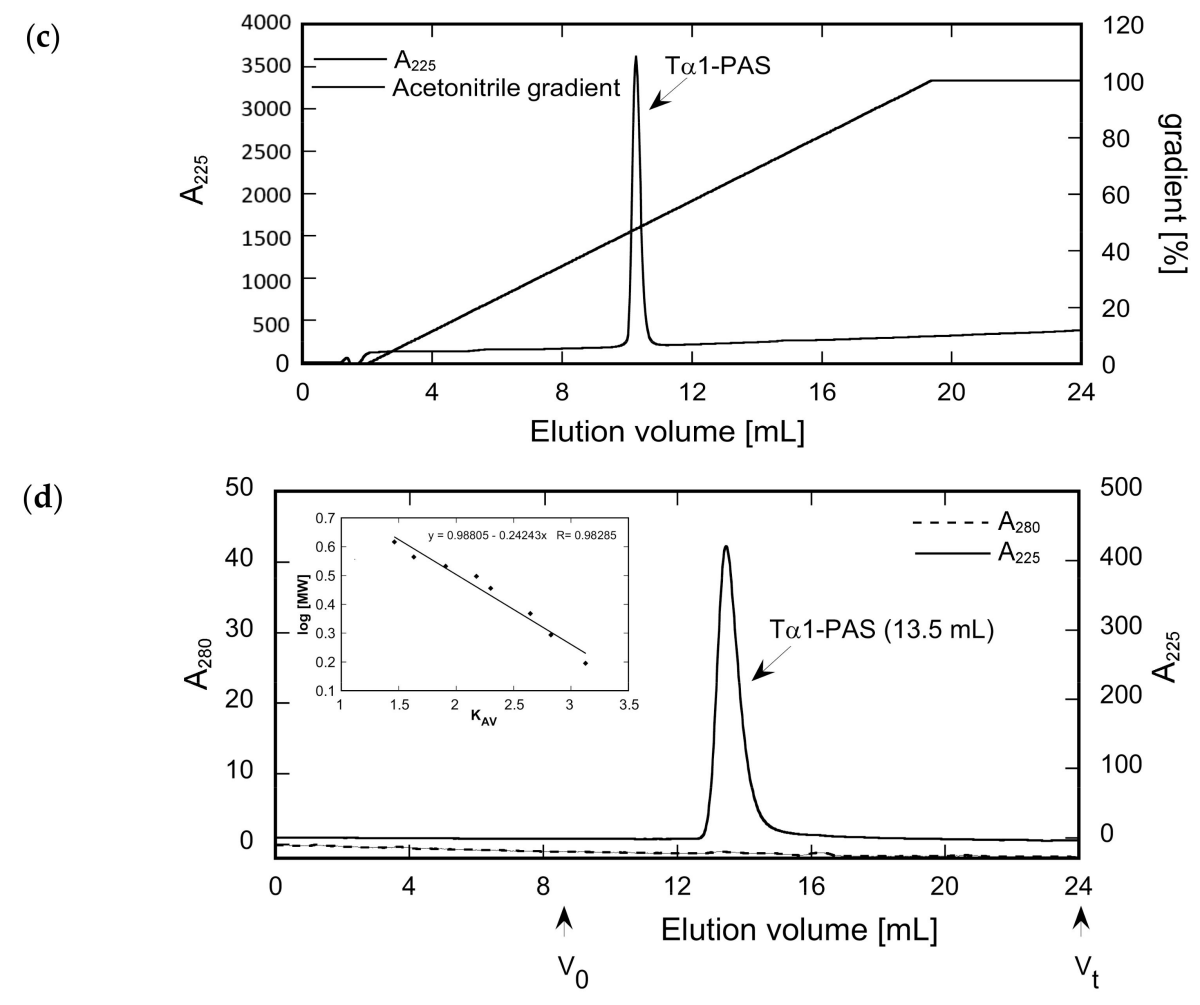
Analysis of Tα1-PAS N-acetylation, peptide purity, and hydrodynamic volume. (**a**) The deconvoluted ESI-MS spectrum of the Tα1-PAS flow-through fraction after the subtractive AEX step (see text) shows a molecular mass of 52,734.56 Da, corresponding to N-acetylated Tα1-PAS (calc. mass: 52,734.56 Da). (**b**) ESI-MS analysis of the bound and eluted peptide fraction from the same chromatography reveals a molecular mass of 52,692.37 Da, which matches the calculated mass of non-acetylated Tα1-PAS (52,692.54 Da). (**c**) Reverse-phase chromatography of the flow-through fraction indicates a purity > 96% for N-acetylated Tα1-PAS. (**d**) Analytical SEC of the finally purified Tα1-PAS in the presence of phosphate-buffered saline (PBS) results in a single peak with an elution volume of 13.5 mL. Comparison with a half-logarithmic calibration curve (inset) indicates an expanded hydrodynamic volume with an apparent molecular size of 557 kDa.

**Figure 3:**
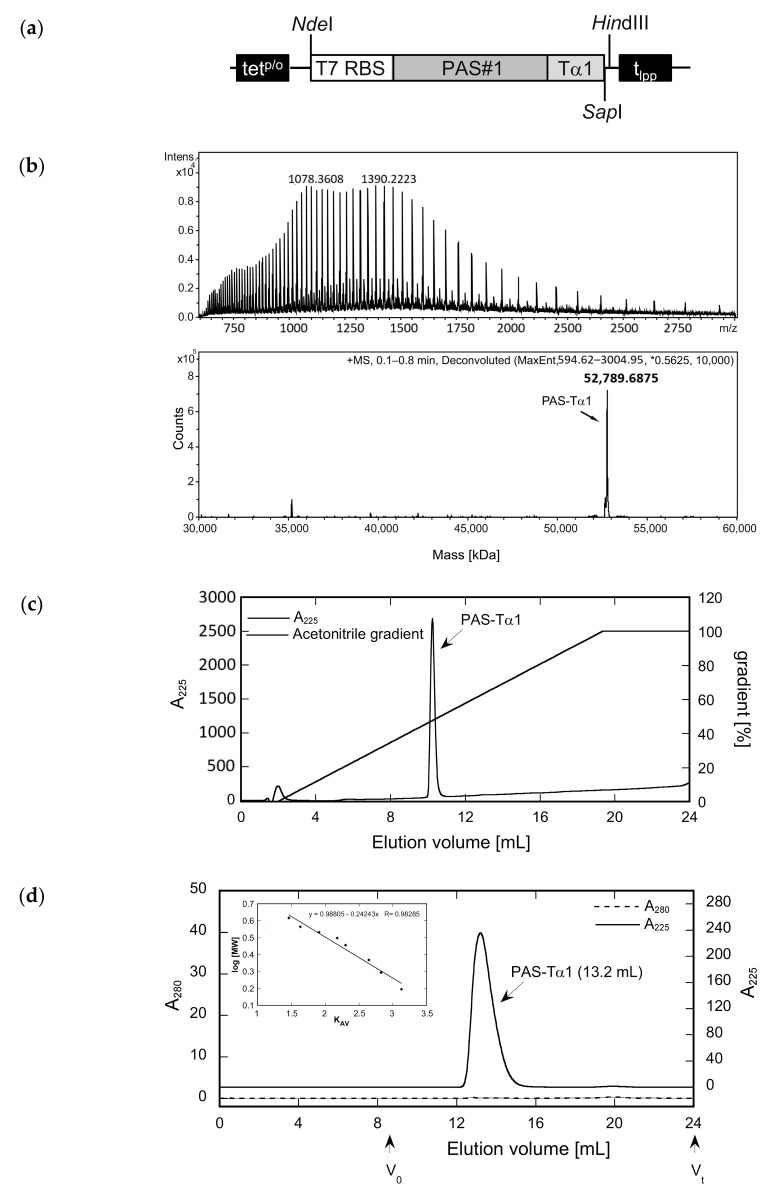
Preparation of an N-terminally PASylated version of the Tα1 peptide and analysis of its integrity, purity, and hydrodynamic volume. (**a**) Expression vector for the bacterial production of PAS-Tα1. The gene cassette cloned on pASK75 comprises the T7 RBS, the coding region for a 601-residue PAS#1 sequence preceded by a start methionine and a proline codon, the human Tα1 sequence, and, finally, t_lpp_. (**b**) The deconvoluted ESI-MS spectrum of PAS-Tα1 purified from the *E. coli* cell extract by subtractive AEX and CEX (see text) reveals a molecular mass of 52,789.69 Da, which matches the calculated mass for PAS-Tα1 with a fully processed start methionine (52,789.66 Da). (**c**) Reverse-phase chromatography indicates a purity > 96% for PAS-Tα1. (**d**) Analytical SEC of the purified PAS-Tα1 in PBS results in a single peak with an elution volume of 13.2 mL. Comparison with a half-logarithmic calibration curve (inset) indicates a strongly expanded hydrodynamic volume with an apparent molecular size of 665 kDa.

The protein solutions were dialyzed against a citrate buffer at pH 3.0 and subsequently applied to a strong cation exchange (CEX) column, which resulted in a bound fraction for PAS-Tα1, whereas both a flow-through fraction and a bound fraction were observed for Tα1-PAS (Figure 2). Electrospray ionization mass spectrometry (ESI-MS) analysis of Tα1-PAS in the flow-through revealed a molecular mass of 52,734.56 Da (Figure 2a), which exactly matches the calculated mass for the N-terminally acetylated gene product (52,734.56 Da). In this case, the start methionine of Tα1-PAS (followed by a Ser residue) was fully processed, presumably by the bacterial methionine aminopeptidase [39], then followed by N-terminal acetylation via RimJ. In contrast, the column-bound peptide fraction, which was eluted using a salt concentration gradient, showed a molecular mass of 52,692.38 Da (Figure 2b), which corresponds to the calculated mass for the non-acetylated processed polypeptide (52,692.54 Da) accompanied by some minor peaks below 40 kDa, most likely due to residual host cell impurities. Accordingly, this CEX step enabled separation of the desired N-acetylated Tα1-PAS from its non-acetylated precursor as a result of a single charge difference. In comparison, the fully column-bound non-acetylated PAS-Tα1 showed a single molecular mass of 52,789.8 Da (Figure 3) corresponding to the intact peptide, again, lacking the start methionine.

Both PASylated peptide preparations had a purity > 96% as indicated by reverse-phase chromatography (Figure 2c and Figure 3c). For Tα1-PAS, we performed a final AEX polishing step, which also allowed concentration of the peptide. At pH 10, the acetylated Tα1-PAS bound to a strong AEX resin and eluted as a homogenous peak in a salt concentration gradient. The endotoxin content of this fraction was very low, with < 0.1 EU/mg, and the final yield was 15 mg acetylated Tα1-PAS per 1 L bacterial culture. In comparison, the final yield of the fully column-bound (non-acetylated) PAS-Tα1 reached 50 mg per 1 L bacterial culture after CEX chromatography and, again, the endotoxin content was below 0.1 EU/mg. Analytical size exclusion chromatography (SEC) of both PASylated peptide versions revealed a single symmetric peak without any signs of aggregation or truncation (Figure 2d and Figure 3d). The N-terminally acetylated Tα1-PAS eluted at 13.5 mL (bed volume: 24 mL), whereas PAS-Tα1 eluted at 13.2 mL, thus indicating apparent molecular sizes of 557 kDa and 665 kDa, respectively. This is more than 10 times (Tα1-PAS) or even 12 times (PAS-Tα1) larger than the true molecular mass of both PASylated peptides (52.7 kDa), which demonstrates the huge expansion of the hydrodynamic molecular volume caused by the random-coil structure of the PAS polymer, in line with previous observations [35].

### 2.3. PASylation Strongly Prolongs Tα1 Pharmacokinetics in Rats

**Figure 4:**
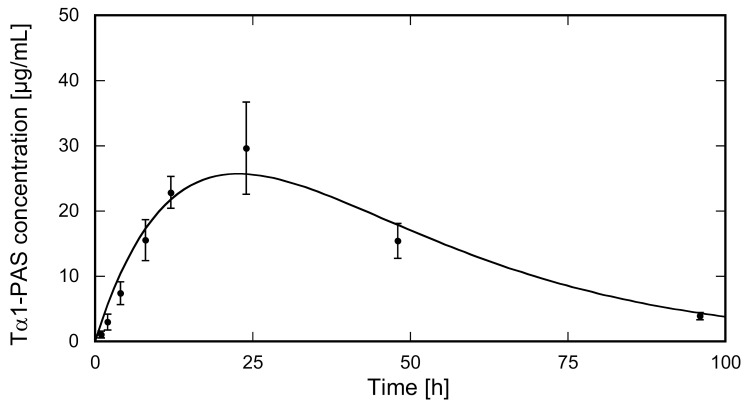
PK study of N-acetylated Tα1-PAS in rats. Tα1-PAS was subcutaneously injected at a dose of 3.4 mg/kg b.w. into female Wistar rats (*N* = 5). The concentration of the fusion protein in plasma was quantified using sandwich ELISA. Data were plotted against the sampling time post injection (p.i.) and fitted using a one-compartment model. The PK profile shows distinct absorption and elimination phases (for PK parameters, see Table 1).

To mimic the clinically approved route of Zadaxin™ administration, the N-acetylated Tα1-PAS was injected subcutaneously into the dorsal area of rats (*N* = 5). The injected dose of 3.4 mg/kg Tα1-PAS was well tolerated without any drug-related adverse events or significant changes in body weight. The Tα1-PAS plasma levels at various sampling times were analyzed using a quantitative sandwich ELISA developed to detect only Tα1-PAS and no endogenous rat Tα1, which shares 100% sequence identity with the human peptide. The pharmacokinetic (PK) profile of Tα1-PAS (Figure 4) exhibited a typical curve according to the Bateman function [40], with a C_max_ of 25.6 ± 4.4 mg/L at t_max_ = 22.7 ± 1.1 h. Curve fitting with the WinNonlin software revealed a drastically extended terminal half-life of 15.9 ± 0.9 h, which is more than 8-fold longer than the one for the native peptide (τ_1/2_ = 1.9 h) published for rats [41]. The strong impact of PASylation on the PK profile is also reflected by other parameters such as the large area under the curve (AUC) and slow clearance (CL) (Table 1).

## 3. Discussion

The present data demonstrate that the PASylation technology can solve two major problems of a peptide drug: (i) instability in the expression host and (ii) low bioactivity due to fast renal clearance. Typically, small peptides, if expressed in a soluble state in *E. coli*, are quickly degraded by host cell proteases [42] and, thus, require production as larger fusion proteins as well as subsequent release by site-specific cleavage in vitro. Here, fusion with a random-coil forming PAS sequence of ~600 amino acids prevented proteolytic degradation and allowed cost-efficient one-step production of the intact fusion protein in *E. coli* without the need of additional processing steps.

Of note, in contrast to classical approaches, which involve the use of an insoluble fusion partner, for example, an α-galactosidase fragment in the case of insulin [43], to provoke formation of inclusion bodies and protect the gene product from intracellular proteolysis, the PASylated Tα1 peptide was recovered as a soluble fusion protein, thus allowing direct purification from the cell extract without solubilization steps. Furthermore, the C-terminally attached PAS moiety was compatible with N-terminal acetylation by RimJ as confirmed by ESI-MS. Yields of our expression study at the research scale reached 15 mg purified acetylated Tα1-PAS and even 50 mg PAS-Tα1 per 1 L bacterial culture (at an optical density (OD) of 3), which surpasses the reported yield of a bacterially produced (yet probably non-acetylated and non-glycosylated) Tα1-Fc fusion protein (16 mg/L) [44].

Still, there is considerable opportunity of improvement for both versions of the PASylated peptide by optimizing the expression plasmid and the production process, including high cell density fermentation under controlled feeding conditions. Apart from that, the absence of a prominent band for the co-expressed enzyme RimJ in SDS-PAGE despite approximately 50% N-terminal acetylation of Tα1-PAS also indicates room for amelioration. Optimization of the ribosome-binding site preceding the RimJ cistron or changing the order within the bicistronic operon should boost biosynthesis of the enzyme and result in increased yield of the N-terminally acetylated Tα1-PAS. Finally, the use of a high-efficiency secretory bacterial expression system such as ESETEC [45] or CORYNEX [46,47] should lead to higher product titers as previously demonstrated for other PASylated fusion proteins such as PASylated human growth hormone (hGH) [48]. The yield of functional PAS-hGH was more than 100-fold higher with the ESETEC system than in a conventional laboratory strain of *E. coli*, reaching several grams per liter culture.

Attachment of the PAS sequence at either end of the Tα1 peptide increased the hydrodynamic volume by more than an order of magnitude as shown by SEC. This strongly expanded molecular size resulted in a plasma half-life in rats of around 16 h after a subcutaneous injection of Tα1-PAS, which is more than an 8-fold increase compared to the unmodified peptide drug (1.9 h) [41]. Based on in vitro cell culture assays with human serum albumin fusion proteins [49], both the C-terminus and the N-terminus of Tα1 should be permissible to modification while retaining bioactivity. This was also shown in animal tumor models for C-terminal fusion with an immunoglobulin Fc fragment [41], an internalizing arginylglycylaspartic acid peptide iRGD [50], and thymopentin [51]. However, in the case of Tα1, some caution is appropriate regarding the significance of in vitro cell culture assays as its mode of action is complex and involves different receptors provoking multiple biological effects on various cell types [52]. Second, a prolonged circulation in the body as demonstrated here via application of the PASylation technology influences both binding kinetics and bioactivity, which is not reflected in vitro. While attachment of large macromolecules, such as PAS polypeptides or albumin, but also PEG [53], can lead to lower receptor association rates for bioactive peptides or proteins, this is usually overcompensated by the drastically prolonged in vivo half-life, which results in a strongly enhanced bioactivity as demonstrated for multiple PASylated biopharmaceuticals [37,54]. In the case of Tα1, superior effects in preclinical cancer models due to prolonged circulation were recently demonstrated for a Tα1-Fc fusion protein [41,44]. Such studies would be the obvious next step to investigate enhanced in vivo bioactivity of PASylated Tα1, and it will be interesting to see whether its N-terminally or C-terminally PASylated version performs better.

In comparison with other published approaches to prolong the circulation of Tα1, the measured terminal half-life of Tα1-PAS is even longer than the value of around 8.2 h reported for a corresponding PEGylated peptide after an intravenous injection into the tail vein of Sprague–Dawley rats [55]. This modified Tα1 was prepared by chemically coupling a 5 kDa methoxypolyethylene glycol maleimide via an engineered N-terminal Cys residue. Of note, according to the rules of allometric scaling, a much longer half-life in the range of several days can be expected for Tα1-PAS in humans [56,57], which would allow weekly dosing while achieving a lasting pharmacological effect. According to the prescription information for Zadaxin™ to treat chronic hepatitis B, the recommended dose for the monotherapy, or the combination therapy with interferon, is a 1.6 mg subcutaneous injection twice weekly over 6 months (52 doses). Consequently, weekly or biweekly injections would considerably decrease patient burden and improve compliance.

Moreover, a more continuous plasma level above the minimum effective dose due to slower clearance, as illustrated by the drastically increased AUC, should boost in vivo efficacy and open new treatment perspectives. Especially in preclinical animal models, which typically suffer from a much quicker drug clearance compared to humans [37] owing to their smaller body size, a long-acting Tα1 should lead to more convincing pharmacodynamic (PD) effects and pave the way for biopharmaceutical development for novel indications such as cystic fibrosis [18], HIV-1 infection [58], sepsis [20], or cancer [59]. For example, cancer studies have demonstrated that high doses of the conventional peptide are required to achieve antitumor activity [60].

The beneficial application of the PASylation technology demonstrated in this work can be transferred to other peptides. There are more than 7000 naturally occurring peptides covering a wide range of physiological functions [61], including many peptides with proven therapeutic potential, which could profit from the presented approach. Examples are therapeutically active peptides such as thymosin beta 4 [62,63], the C-type natriuretic peptide (CNP) [64], human parathyroid hormone (PTH) [65], relaxin [66], or glucagon-like peptide-1 (GLP-1) and its analogs [67]. Today, it is generally recognized that intrinsic weaknesses of this drug class, such as poor stability and short circulating plasma half-life, need to be addressed in order to transform peptides into efficacious medicines [61]. The approach described here, N- or C-terminal PASylation, optionally combined with acetylation, solves both. On the one hand, N-terminal acetylation protects peptides from proteolytic degradation by exoproteases, for example, dipeptidyl peptidase-4 (DPP-IV), as shown for N-terminally acetylated GLP-1 [68]. On the other hand, PASylation increases the hydrodynamic volume of the peptide above the pore size of the glomerular basement membrane, hence retarding kidney filtration and prolonging the pharmacodynamic effect of its fusion partner [31].

Furthermore, PASylation can serve as a linker to join two peptides [69]. This is of particular interest if both entities act synergistically, for example, GLP-1 and GIP [70] or Tα1 and GM-CSF [71]. Alternatively, the biologically active protein/peptide can be linked via the PAS sequence to a targeting domain such as the arginylglycylaspartic acid peptide RGD peptide which binds to integrins αVβ3 and αVβ5 in order to enhance tumor penetration and accumulation. In fact, fusion of the short-acting Tα1 with the tumor-targeting iRGD peptide using a Gly_4_ linker has recently demonstrated enhanced antitumor activity [50]. In contrast to synthetic PEG linkers, which are commonly used for bioconjugations, the recombinant PAS sequence exhibits a precisely defined size and is biodegradable [72]. The PAS polypeptide itself is stable in blood plasma but quickly degraded by intracellular enzymes, thus avoiding organ accumulation [31], a well-known effect for PEGylation [73]. Moreover, PAS sequences are non-immunogenic in animals [31,74] and offer a one-step production of PASylated peptides in various commercially scalable expression systems including bacteria, yeasts, or mammalian cells [35,48], which would even allow the preparation of peptides carrying posttranslational modifications.

## 4. Materials and Methods

### 4.1. Construction of the Expression Plasmid

A bicistronic operon for the simultaneous expression of human Tα1 (UniProt P06454, residues 2–29) and the *E. coli* N-acetyltransferase RimJ (UniProt P0A948) flanked by the restriction sites *Nde*I and *Hin*dIII was prepared using gene synthesis (Thermofisher Scientific, Regensburg, Germany). To this end, the coding sequence of human Tα1 was codon-optimized for expression in *E. coli* and linked to the RimJ structural gene via the nucleotide sequence GCCTGAAGAGCAGAAAATAAA comprising a “GCC” alanine codon, the opal stop codon “TGA”, a *Sap*I restriction site for insertion of the PAS gene cassette, and a ribosome-binding site (RBS). The entire gene fragment was subcloned via *Nde*I and *Hin*dIII on a derivative of pASK75 [36] for cytoplasmic expression under control of the tetracycline promoter/operator (tet^p/o^). Subsequently, a substantially non-repetitive sequence-verified PAS gene cassette [34] encoding a PAS#1 polypeptide of 601 amino acids was inserted via the *Sap*I restriction site to create the expression vector for the C-terminally PASylated thymosin α1 (Tα1-PAS), pASK75-Tα1-PAS#1(600)/RimJ. To construct a plasmid for the bacterial production of an N-terminally PASylated Tα1 (PAS-Tα1), the synthetic gene of human Tα1 was amplified via PCR using the primers THY-For (5’-AGCTCTTCTGCCAGTGATGCAGCAGTTGATACC) and THY-Rev (5’-GCTCAAGCTTAGTTCTCGGCTTCTTCCAC). The PCR product was digested with *Sap*I and *Hin*dIII and inserted via these restriction sites downstream of the PAS#1(600) sequence preceded by Met and Pro encoded on a suitable pASK75 plasmid derivative.

### 4.2. Bacterial Production of Recombinant PASylated Tα1

The BL21 derivative *E. coli* strain NEBexpress^®^ (New England Biolabs, Frankfurt am Main, Germany) was transformed with the plasmids pASK75-Tα1-PAS#1(600)/RimJ or pASK75-MP-PAS#1(600)-Tα1 from above. Two milliliters of Terrific Broth supplemented with 100 µg/mL ampicillin (TB_amp_) was inoculated with a single colony and grown overnight at 37 °C, 170 rpm. This overnight culture was used to inoculate a 50 mL preculture, which was grown under the same conditions until OD_550_ = 1.1 was reached. Subsequently, the 50 mL preculture was transferred into 2 L TB_amp_ and incubated in a 5 L baffled flask at 37 °C and 100 rpm. After reaching OD_550_ = 0.6, the temperature was decreased to 26 °C and the cells were subsequently induced at OD_550_ = 1 with 200 µg/mL aTc for 15 h.

### 4.3. Purification of PASylated Tα1

Bacteria were harvested by centrifugation and disrupted in the presence of 100 mM citric acid using an EmulsiFlex C5 cell homogenizer (Avestin, Mannheim, Germany). After centrifugation for 20 min (39,200× *g*) at 16 °C, the supernatant was filter-sterilized using a 0.45 µm syringe filter (Sartorius, Göttingen, Germany). The clear filtrate was adjusted with ammonium sulfate to 30% saturation, stirred for 30 min at RT, and centrifuged for 30 min (39,200× *g*) at RT. The precipitate was solubilized in subtractive AEX buffer (sAEX; 20 mM Tris/HCl, 1 mM EDTA, pH 8.5), filter-sterilized, and dialyzed twice against a 100-fold volume of sAEX buffer at 4 °C. Subsequent chromatography steps were performed on an ÄKTA Explorer 100 system (GE Healthcare, Freiburg, Germany) operated at a flow rate of 5 mL/min. The dialyzed sample (Tα1-PAS or PAS-Tα1) was first applied to a 5 mL TOYOPEARL NH2-750F column (Tosoh Bioscience, Griesheim, Germany) equilibrated with the sAEX buffer. The flow-through containing the PASylated Tα1 was collected and dialyzed twice against a 100-fold volume of the CEX buffer (20 mM Na-citrate, pH 3.0) at 4 °C. The protein solution was then applied to an 85 mL TOYOPEARL Sulfate-650F column (Tosoh Bioscience) equilibrated with the CEX buffer. PAS-Tα1 quantitatively bound to the column and the pure protein was eluted using a NaCl concentration gradient (0–250 mM over 2 column volumes). In the case of Tα1-PAS, the CEX flow-through fraction contained the N-terminally acetylated protein, whereas the non-acetylated Tα1-PAS stayed bound to the column and could again be eluted in a NaCl concentration gradient. The acetylated Tα1-PAS fraction from the flow-through was dialyzed twice against AEX buffer (20 mM ethanolamine/HCl, pH 10) and then applied to a 100 mL Fractogel^®^ EMD TMAE (M)strong anion exchanger (Merck Millipore, Burlington, MA, USA) equilibrated with the AEX buffer. This time, highly pure acetylated Tα1-PAS was eluted using a NaCl concentration gradient (0–200 mM over 2 column volumes) in the AEX buffer. Both purified PASylated peptides were dialyzed 5 times against a 200-fold volume of water, lyophilized, and stored at −20 °C until further use.

### 4.4. Analytical Size Exclusion Chromatography

Analytical SEC was performed on a 24 mL Superose^®^ 6 10/300 GL column (GE Healthcare) using an ÄKTA Explorer 10 system operated at a flow rate of 0.5 mL/min with PBS as the running buffer. To determine the apparent molecular size, purified PAS-Tα1 or PAS-Tα1 (100 µL) was applied to the column and the elution volume was used for linear interpolation from a half-logarithmic calibration line obtained using the reference proteins thyroglobulin (octamer), thyroglobulin (tetramer), apoferritin, β-amylase, alcohol dehydrogenase (tetramer), transferrin, ovalbumin, and carboanhydrase as well as blue dextran (all from Merck, Darmstadt, Germany).

### 4.5. Endotoxin Quantification

Tα1-PAS samples were diluted to 1 mg/mL in endotoxin-free water (Veolia Water Technologies, Celle, Germany), heated to 90 °C for 5 min, and, after cooling down to RT, bacterial endotoxins were quantified using an Endosafe^®^ system (Charles River Laboratories, Wilmington, MA, USA) with an FDA-licensed PTS™ cartridge (10–0.1 EU/mL sensitivity).

### 4.6. Western Blot Analysis

The whole cell protein extract was applied to a 4–12% SurePAGE™ Bis–Tris gradient gel (Genscript, Piscataway, NJ, USA) with 3-(N-morpholino)propanesulfonic acid (MOPS) as running buffer and electrotransferred onto a nitrocellulose membrane using an iBlot 1 dry blotting system (ThermoFisher, Waltham, MA, USA). After washing 3 times with PBS supplemented with 0.1% *v/v* Tween 20 (PBS/T), the membrane was incubated with 1 µg/mL anti-PAS antibody in PBS/T for 1 h at RT. The membrane was washed 3 times with PBS/T and incubated with a 1:5000 dilution of a goat anti-mouse IgG (Fc-specific) alkaline phosphatase (AP) conjugate (Merck) for 1 h. After washing twice with PBS/T and twice with PBS, the blot was developed by a chromogenic reaction using BCIP (37.5 µg/mL) and NBT (150 µg/mL) in alkaline phosphatase buffer (100 mM Tris/HCl, pH 8.8, 100 mM NaCl, 5 mM MgCl_2_).

### 4.7. Reverse-Phase Chromatography (RPC) and ESI Mass Spectrometry

Directly after the CEX chromatography, a 200 µl sample of PAS-Tα1 or PAS-Tα1 was adjusted to 2% *v/v* acetonitrile, 0.1% *v/v* formic acid and applied to a Resource RPC 1 mL column (GE Healthcare) equilibrated with 2% *v/v* acetonitrile, 0.1% *v/v* formic acid. The protein was eluted using a concentration gradient of up to 80% *v/v* acetonitrile, 0.1% *v/v* formic acid over 20 column volumes at a flow rate of 2 mL/min while spectrophotometrically monitoring protein elution at 225 nm. The eluted protein fraction was directly injected into a maXis quadrupole time of flight (Q-TOF) instrument (Bruker Daltonics, Bremen, Germany) operated in the positive ion mode. Raw data was deconvoluted using the MaxEntX algorithm (Bruker Daltonics).

### 4.8. Pharmacokinetic Analysis in Rats

A PK study in female Wistar rats at 8–9 weeks of age was conducted at the Aurigon Toxicological Research Center (ATRC, Dunakeszi, Hungary). Up to 3 animals per cage were housed in a controlled environment at 22 ± 3 °C with a relative humidity of 50 ± 20%, 12 h light and 12 h dark. Endotoxin-free purified Tα1-PAS (3.4 mg/kg) was administered subcutaneously via a single injection into the rat dorsal area. Blood samples (100 µL) were taken from 5 animals each at various time points. Following collection in tubes containing tri-potassium ethylenediaminetetraacetic acid (K3-EDTA) (Greiner Bio-One, Frickenhausen, Germany), samples were centrifuged at room temperature for 10 min (3000× *g*) and the resulting plasma was stored at −15 to −30 °C. Tα1-PAS in these samples was quantified using sandwich ELISA (see below) and the data were analyzed using the Phoenix WinNonlin 6.3 software (Certara, Princeton, NJ, USA) using a one-compartment model assuming 1st order absorption and elimination.

### 4.9. Quantification of Purified Tα1-PAS

Due to the absence of aromatic side chains, PASylated Tα1 does not absorb light at 280 nm. Thus, for reliable quantification via the peptide backbone absorption, an extinction coefficient at 225 nm was determined. To this end, highly pure Tα1-PAS was lyophilized from water, weighed, and dissolved in a defined volume of water. The concentration of this Tα1-PAS solution was additionally quantified by measuring the backbone peptide groups using UV absorption at 205 nm using a generic calculated extinction coefficient [75], which led to a fair agreement (±6%). The absorbance at 225 nm was measured for various dilutions of the gravimetrically prepared peptide stock solution and plotted against the concentration. The linear range was fitted by a straight line, resulting in an extinction coefficient of 253,057 M^−1^⋅cm^−1^. While most buffers strongly absorb light at 205 nm, such a spectral overlap is usually absent at 225 nm.

### 4.10. ELISA Quantification of PASylated Tα1

A 96-well Nunc Maxisorb ELISA plate (ThermoFisher) was coated with 100 µg/mL anti-PAS antibody in PBS at 4 °C overnight. After washing twice with PBS/T, free binding sites were blocked with 3% *w/v* bovine serum albumin (BSA) in PBS/T at room temperature for 1 h. After washing 3 times with PBS/T, the rat plasma samples were applied in dilution series in PBS/T supplemented with 0.5% (*v/v*) plasma from an untreated animal (to maintain a constant proportion of rat plasma constituents). In the same manner, a standard curve was prepared using dilution series of purified Tα1-PAS (used for spiking rat plasma) at defined concentrations. After incubation for 1 h at RT, wells were washed 3 times with PBS/T. To detect bound Tα1-PAS, wells were incubated for 1 h with 50 µl of a 1 µg/mL PBS/T solution of the second anti-PAS antibody conjugated with alkaline phosphatase using the Lightning-Link alkaline phosphatase antibody labeling kit (BioTechne, Wiesbaden, Germany). After washing twice with PBS/T and twice with PBS, the enzymatic activity was detected using p-nitrophenyl phosphate (0.5 mg/mL). To this end, the plate was incubated for 20 min at 30 °C, the absorbance was measured at 405 nm using a SpectraMax M5e microtiter plate reader (Molecular Devices, Sunnyvale, CA, USA) and the Tα1-PAS concentrations in rat plasma samples were quantified by comparison with the standard curve.

## Figures and Tables

**Table 1:** Pharmacokinetic properties of Tα1-PAS in rats.

Parameter	Tα1-PAS
C_max_ (mg/L)	25.6 ± 4.4
t_max_ (h)	22.7 ± 1.1
AUC_0-∞_ (h µg/mL)	1586.7 ± 295.1
τ_1/2_α (h)	15.7 ± 0.8
τ_1/2_β (h)	15.9 ± 0.9
CL (mL/h/kg)	2.2 ± 0.4

## Data Availability

The data presented in this study are available on request from the corresponding authors.

## Abbreviations

AEX	Anion exchange
AP	Alkaline phosphatase
aTc	Anhydrotetracycline hydrochloride
AUC	Area und the curve
BCIP	5-Bromo-4-chloro-3-indoxyl phosphate
BSA	Bovine serum albumin
CEX	Cation exchange
CL	Clearance
C_max_	Maximum (or peak) serum concentration
CNP	C-type natriuretic peptide
CORYNEX	*Corynebacterium glutamicum* protein expression system
COVID-19	Severe acute respiratory syndrome coronavirus 2
CTLA-4	Cytotoxic T-lymphocyte-associated protein 4
CV	Column volume
DCs	Dendritic cells
DPP-IV	Dipeptidyl peptidase-4
ELISA	Enzyme-linked immunosorbent assay
ESETEC	*E. coli* secretion technology
ESI-MS	Electrospray ionization mass spectrometry
FPLC	Fast protein liquid chromatography
GCC	Glucagon
GIP	Glucose-dependent insulinotropic polypeptide
GLP-1	Glucagon-like peptide 1
GM-CSF	Granulocyte-macrophage colony-stimulating factor
GSH	γ-L-Glutamyl-L-cysteinyl-glycine
HBV	Hepatitis B virus
HCV	Hepatitis C virus
hGH	Human growth hormone
hIFN-α	Human interferon 1
HIV-1	Human immunodeficiency virus I
iRGD	Internalizing arginylglycylaspartic acid peptide
K3-EDTA	Tri-potassium ethylenediaminetetraacetic acid
MHC	Major histocompatibility complex
MOPS	3-(N-morpholino)propanesulfonic acid
NBT	Nitro blue tetrazolium
OD	Optical density at 550 nm
PAS	Proline/alanine-rich sequence
PAS-Tα1	N-terminally PASylated Tα1
PBS	Phosphate-buffered saline
PD	Pharmacodynamics
PEG	Polyethylene glycol
p.i.	Post injection
PK	Pharmacokinetics
ProTα	Prothymosin alpha
PTH	Parathyroid hormone
Q-TOF	Quadrupole time of flight
RBS	Ribosome-binding site
RGD	Arginylglycylaspartic acid
RimJ	Ribosomal-protein-S5-alanine N-acetyltransferase
RPC	Reverse phase chromatography
RT	Room temperature
SEC	Size exclusion chromatography
SDS-PAGE	Sodium dodecyl sulfate polyacrylamide gel electrophoresis
τ_1/2_α	Half-life of distribution
τ_1/2β_	Elimination half-life
Tα1	Thymosin alpha 1
Tα1-PAS	C-terminally PASylated Thymosin alpha 1
tet^p/o^	Tetracycline promoter/operator
Th	T helper cell
t_lpp_	Lipoprotein terminator
TLR-2	Toll-like receptor 1
TLR-9	Toll-like receptor 2
TMAE	Trimethylaminoethyl
t_max_	Time to reach C_max_

## References

[1] Goldstein A.L., Low T.L., McAdoo M., McClure J., Thurman G.B., Rossio J., Lai C.Y., Chang D., Wang S.S., Harvey C. (1977). Thymosin alpha1: Isolation and sequence analysis of an immunologically active thymic polypeptide. Proc. Natl. Acad. Sci. USA.

[2] Hoch K., Volk D.E. (2016). Structures of thymosin proteins. Vitam. Horm..

[3] Gast K., Damaschun H., Eckert K., Schulze-Forster K., Maurer H.R., Muller-Frohne M., Zirwer D., Czarnecki J., Damaschun G. (1995). Prothymosin alpha: A biologically active protein with random coil conformation. Biochemistry.

[4] Sarandeses C.S., Covelo G., Diaz-Jullien C., Freire M. (2003). Prothymosin alpha is processed to thymosin alpha 1 and thymosin alpha 11 by a lysosomal asparaginyl endopeptidase. J. Biol. Chem..

[5] Camerini R., Garaci E. (2015). Historical review of thymosin alpha 1 in infectious diseases. Expert Opin. Biol. Ther..

[6] Romani L., Bistoni F., Gaziano R., Bozza S., Montagnoli C., Perruccio K., Pitzurra L., Bellocchio S., Velardi A., Rasi G. (2004). Thymosin alpha 1 activates dendritic cells for antifungal Th1 resistance through toll-like receptor signaling. Blood.

[7] King R., Tuthill C. (2016). Immune modulation with thymosin alpha 1 treatment. Vitam. Horm..

[8] Baumann C.A., Badamchian M., Goldstein A.L. (1997). Thymosin alpha 1 antagonizes dexamethasone and CD3-induced apoptosis of CD4+ CD8+ thymocytes through the activation of cAMP and protein kinase C dependent second messenger pathways. Mech. Ageing Dev..

[9] Giuliani C., Napolitano G., Mastino A., Di Vincenzo S., D’Agostini C., Grelli S., Bucci I., Singer D.S., Kohn L.D., Monaco F. (2000). Thymosin-alpha1 regulates MHC class I expression in FRTL-5 cells at transcriptional level. Eur. J. Immunol..

[10] Garaci E., Pica F., Serafino A., Balestrieri E., Matteucci C., Moroni G., Sorrentino R., Zonfrillo M., Pierimarchi P., Sinibaldi-Vallebona P. (2012). Thymosin α1 and cancer: Action on immune effector and tumor target cells. Ann. N. Y. Acad. Sci..

[11] Palamara A., Bue M., Savini P. Thymosin alpha 1 inhibits Sendai virus replication: Involvement of intracellular redox state. Proceedings of the 6th International Expert Forum of Immunotherapy and Gene Therapy.

[12] Moody T.W., Fagarasan M., Zia F., Cesnjaj M., Goldstein A.L. (1993). Thymosin alpha 1 down-regulates the growth of human non-small cell lung cancer cells in vitro and in vivo. Cancer Res..

[13] Sgarbanti R., Nencioni L., Amatore D., Coluccio P., Fraternale A., Sale P., Mammola C.L., Carpino G., Gaudio E., Magnani M. (2011). Redox regulation of the influenza hemagglutinin maturation process: A new cell-mediated strategy for anti-influenza therapy. Antioxid. Redox Signal..

[14] Tuthill C.W., King R.S. (2013). Thymosin alpha 1—A peptide immune modulator with a broad range of clinical applications. Clin. Exp. Pharmacol..

[15] Costantini C., Bellet M.M., Pariano M., Renga G., Stincardini C., Goldstein A.L., Garaci E., Romani L. (2019). A reappraisal of thymosin alpha1 in cancer therapy. Front. Oncol..

[16] Danielli R., Cisternino F., Giannarelli D., Calabro L., Camerini R., Savelli V., Bova G., Dragonetti R., Di Giacomo A.M., Altomonte M. (2018). Long-term follow up of metastatic melanoma patients treated with Thymosin alpha-1: Investigating immune checkpoints synergy. Expert Opin. Biol. Ther..

[17] Renga G., Bellet M.M., Pariano M., Gargaro M., Stincardini C., D’Onofrio F., Mosci P., Brancorsini S., Bartoli A., Goldstein A.L. (2020). Thymosin α1 protects from CTLA-4 intestinal immunopathology. Life Sci. Alliance.

[18] Romani L., Oikonomou V., Moretti S., Iannitti R.G., D’Adamo M.C., Villella V.R., Pariano M., Sforna L., Borghi M., Bellet M.M. (2017). Thymosin alpha1 represents a potential potent single-molecule-based therapy for cystic fibrosis. Nat. Med..

[19] Severa M., Zhang J., Giacomini E., Rizzo F., Etna M.P., Cruciani M., Garaci E., Chopp M., Coccia E.M. (2019). Thymosins in multiple sclerosis and its experimental models: Moving from basic to clinical application. Mult. Scler. Relat. Disord..

[20] Pei F., Guan X., Wu J. (2018). Thymosin alpha 1 treatment for patients with sepsis. Expert Opin. Biol. Ther..

[21] Lin H.Y. (2020). The severe COVID-19: A sepsis induced by viral infection? And its immunomodulatory therapy. Chin. J. Traumatol..

[22] Meng Z., Wang T., Li C., Chen X., Li L., Qin X., Li H., Luo J. (2020). An experimental trial of recombinant human interferon alpha nasal drops to prevent coronavirus disease 2019 in medical staff in an epidemic area. medRxiv.

[23] Liu Y., Pang Y., Hu Z., Wu M., Wang C., Feng Z., Mao C., Tan Y., Chen L., Li M. (2020). Thymosin alpha 1 (Talpha1) reduces the mortality of severe COVID-19 by restoration of lymphocytopenia and reversion of exhausted T cells. Clin. Infect. Dis..

[24] Toniolo C., Bonora G.M., Heimer E.P., Felix A.M. (1987). Structure, solubility and reactivity of peptides. A conformational study of two protected key intermediates from a large-scale synthesis of thymosin alpha 1. Int. J. Pept. Protein Res..

[25] Schmidt M., Toplak A., Rozeboom H.J., Wijma H.J., Quaedflieg P., van Maarseveen J.H., Janssen D.B., Nuijens T. (2018). Design of a substrate-tailored peptiligase variant for the efficient synthesis of thymosin-alpha1. Org. Biomol. Chem..

[26] Liu B., Gong X., Chang S., Sun P., Wu J. (2013). Generation of mature Nalpha-terminal acetylated thymosin alpha 1 by cleavage of recombinant prothymosin alpha. Sci. World J..

[27] Zhou L., Lai Z.T., Lu M.K., Gong X.G., Xie Y. (2008). Expression and hydroxylamine cleavage of thymosin alpha 1 concatemer. J. Biomed. Biotechnol..

[28] Ren Y., Yao X., Dai H., Li S., Fang H., Chen H., Zhou C. (2011). Production of Nalpha-acetylated thymosin alpha1 in Escherichia coli. Microb. Cell Fact..

[29] Chen P.F., Zhang H.Y., Fu G.F., Xu G.X., Hou Y.Y. (2005). Overexpression of soluble human thymosin alpha 1 in Escherichia coli. Acta Biochim. Biophys. Sin..

[30] Rost K.L., Wierich W., Masayuki F., Tuthill C.W., Horwitz D.L., Herrmann W.M. (1999). Pharmacokinetics of thymosin alpha1 after subcutaneous injection of three different formulations in healthy volunteers. Int. J. Clin. Pharmacol. Ther..

[31] Schlapschy M., Binder U., Börger C., Theobald I., Wachinger K., Kisling S., Haller D., Skerra A. (2013). PASylation: A biological alternative to PEGylation for extending the plasma half-life of pharmaceutically active proteins. Protein Eng. Des. Sel..

[32] Fang H., Zhang X., Shen L., Si X., Ren Y., Dai H., Li S., Zhou C., Chen H. (2009). RimJ is responsible for N(alpha)-acetylation of thymosin alpha1 in Escherichia coli. Appl. Microbiol. Biotechnol..

[33] Breibeck J., Skerra A. (2018). The polypeptide biophysics of proline/alanine-rich sequences (PAS): Recombinant biopolymers with PEG-like properties. Biopolymers.

[34] Binder U., Skerra A. (2017). PASylation^®^: A versatile technology to extend drug delivery. Curr. Opin. Colloid Int..

[35] Gebauer M., Skerra A. (2018). Prospects of PASylation^@^ for the design of protein and peptide therapeutics with extended half-life and enhanced action. Bioorg. Med. Chem..

[36] Skerra A. (1994). Use of the tetracycline promoter for the tightly regulated production of a murine antibody fragment in Escherichia coli. Gene.

[37] Morath V., Bolze F., Schlapschy M., Schneider S., Sedlmayer F., Seyfarth K., Klingenspor M., Skerra A. (2015). PASylation of murine leptin leads to extended plasma half-life and enhanced in vivo efficacy. Mol. Pharm..

[38] Gattiker A., Duvaud S., Wilkins M.R., Appel R.D., Bairoch A., Walker J.M. (2005). Protein Identification and Analysis Tools on the ExPASy Server. The Proteomics Protocols Handbook.

[39] Xiao Q., Zhang F., Nacev B.A., Liu J.O., Pei D. (2010). Protein N-terminal processing: Substrate specificity of Escherichia coli and human methionine aminopeptidases. Biochemistry.

[40] Garrett E.R. (1994). The Bateman function revisited: A critical reevaluation of the quantitative expressions to characterize concentrations in the one compartment body model as a function of time with first-order invasion and first-order elimination. J. Pharmacokinet. Biopharm..

[41] Wang F., Yu T., Zheng H., Lao X. (2018). Thymosin alpha1-Fc modulates the immune system and down-regulates the progression of melanoma and breast cancer with a prolonged half-life. Sci. Rep..

[42] Wegmüller S., Schmid S. (2014). Recombinant peptide production in microbial cells. Curr. Org. Chem..

[43] Goeddel D.V., Kleid D.G., Bolivar F., Heyneker H.L., Yansura D.G., Crea R., Hirose T., Kraszewski A., Itakura K., Riggs A.D. (1979). Expression in Escherichia coli of chemically synthesized genes for human insulin. Proc. Natl. Acad. Sci. USA.

[44] Shen X., Li Q., Wang F., Bao J., Dai M., Zheng H., Lao X. (2018). Generation of a novel long-acting thymosin alpha1-Fc fusion protein and its efficacy for the inhibition of breast cancer in vivo. Biomed. Pharmacother..

[45] Mücke M.O.R., Leonhartsberger S. (2009). E. coli secretion technologies enable production of high yields of active human antibody fragments. BioProcess Int..

[46] Kikuchi Y., Date M., Yokoyama K., Umezawa Y., Matsui H. (2003). Secretion of active-form Streptoverticillium mobaraense transglutaminase by Corynebacterium glutamicum: Processing of the pro-transglutaminase by a cosecreted subtilisin-Like protease from Streptomyces albogriseolus. Appl. Environ. Microbiol..

[47] Matsuda Y., Itaya H., Kitahara Y., Theresia N.M., Kutukova E.A., Yomantas Y.A., Date M., Kikuchi Y., Wachi M. (2014). Double mutation of cell wall proteins CspB and PBP1a increases secretion of the antibody Fab fragment from Corynebacterium glutamicum. Microb. Cell. Fact..

[48] Di Cesare S., Binder U., Maier T., Skerra A. (2013). High-yield production of PASylated human growth hormone using secretory *E. coli* technology. BioProcess Int..

[49] Chen J., Li H., Wang T., Sun S., Liu J. (2017). Production of N(alpha)-acetyl Talpha1-HSA through in vitro acetylation by RimJ. Oncotarget.

[50] Lao X., Liu M., Chen J., Zheng H. (2013). A tumor-penetrating peptide modification enhances the antitumor activity of thymosin alpha 1. PLoS ONE.

[51] Gao D., Zhang X., Zhang J., Cao J., Wang F. (2008). Expression of thymosin alpha1-thymopentin fusion peptide in Pichia pastoris and its characterization. Arch. Pharm. Res..

[52] Moretti S., Oikonomou V., Garaci E., Romani L. (2015). Thymosin alpha1: Burying secrets in the thymus. Expert Opin. Biol. Ther..

[53] Kubetzko S., Sarkar C.A., Plückthun A. (2005). Protein PEGylation decreases observed target association rates via a dual blocking mechanism. Mol. Pharmacol..

[54] Powers N.E., Swartzwelter B., Marchetti C., de Graaf D.M., Lerchner A., Schlapschy M., Datar R., Binder U., Edwards C.K., Skerra A. (2020). PASylation of IL-1 receptor antagonist (IL-1Ra) retains IL-1 blockade and extends its duration in mouse urate crystal-induced peritonitis. J. Biol. Chem..

[55] Peng G., Pan X., Hu H., Xu Y., Wu C. (2019). N-terminal site-specific PEGylation enhances the circulation half-life of Thymosin alpha 1. J. Drug Deliv. Sci. Technol..

[56] Caldwell G.W., Masucci J.A., Yan Z., Hageman W. (2004). Allometric scaling of pharmacokinetic parameters in drug discovery: Can human CL, Vss and t1/2 be predicted from in-vivo rat data?. Eur. J. Drug Metab. Pharmacokinet..

[57] Mahmood I. (2005). Interspecies Pharmacokinetic Scaling: Principles and Application of Allometric Scaling.

[58] Matteucci C., Grelli S., Balestrieri E., Minutolo A., Argaw-Denboba A., Macchi B., Sinibaldi-Vallebona P., Perno C.F., Mastino A., Garaci E. (2017). Thymosin alpha 1 and HIV-1: Recent advances and future perspectives. Future Microbiol..

[59] King R.S., Tuthill C. (2015). Evaluation of thymosin alpha 1 in nonclinical models of the immune-suppressing indications melanoma and sepsis. Expert Opin. Biol. Ther..

[60] Garaci E., Pica F., Matteucci C., Gaziano R., D’Agostini C., Miele M.T., Camerini R., Palamara A.T., Favalli C., Mastino A. (2015). Historical review on thymosin alpha1 in oncology: Preclinical and clinical experiences. Expert Opin. Biol. Ther..

[61] Fosgerau K., Hoffmann T. (2015). Peptide therapeutics: Current status and future directions. Drug Discov. Today.

[62] Pipes G.T., Yang J. (2016). Cardioprotection by thymosin beta 4. Vitam. Horm..

[63] Sosne G. (2018). Thymosin beta 4 and the eye: The journey from bench to bedside. Expert Opin. Biol. Ther..

[64] Lumsden N.G., Khambata R.S., Hobbs A.J. (2010). C-type natriuretic peptide (CNP): Cardiovascular roles and potential as a therapeutic target. Curr. Pharm. Des..

[65] Leder B.Z. (2017). Parathyroid hormone and parathyroid hormone-related protein analogs in osteoporosis therapy. Curr. Osteoporos. Rep..

[66] Jelinic M., Marshall S.A., Stewart D., Unemori E., Parry L.J., Leo C.H. (2018). Peptide hormone relaxin: From bench to bedside. Am. J. Physiol. Regul. Integr. Comp. Physiol..

[67] Sharma D., Verma S., Vaidya S., Kalia K., Tiwari V. (2018). Recent updates on GLP-1 agonists: Current advancements & challenges. Biomed. Pharmacother..

[68] John H., Maronde E., Forssmann W.G., Meyer M., Adermann K. (2008). N-terminal acetylation protects glucagon-like peptide GLP-1-(7-34)-amide from DPP-IV-mediated degradation retaining cAMP- and insulin-releasing capacity. Eur. J. Med. Res..

[69] Eggenstein E., Richter A., Skerra A. (2019). FluoroCalins: Engineered lipocalins with novel binding functions fused to a fluorescent protein for applications in biomolecular imaging and detection. Protein Eng. Des. Sel..

[70] Mathiesen D.S., Bagger J.I., Bergmann N.C., Lund A., Christensen M.B., Vilsboll T., Knop F.K. (2019). The effects of dual GLP-1/GIP receptor agonism on glucagon secretion–A review. Int. J. Mol. Sci..

[71] Lan H., Hao Y., Lv Y., Li G., Mo Y., Zheng C., Yuanfa L. (2020). Synergistic effect of a combination of granulocyte macrophage colony-stimulating factor and thymosin α1 on Lewis lung cancer transplanted tumor in mice. Trop. J. Pharm. Res..

[72] Kjeldsen T., Hogendorf W.F.J., Tornoe C.W., Anderson J., Hubalek F., Stidsen C.E., Sorensen J.L., Hoeg-Jensen T. (2020). Dually reactive long recombinant linkers for bioconjugations as an alternative to PEG. ACS Omega.

[73] Fletcher A.M., Tellier P., Douville J., Mansell P., Graziano M.J., Mangipudy R.S., Brodie T.A., Achanzar W.E. (2019). Adverse vacuolation in multiple tissues in cynomolgus monkeys following repeat-dose administration of a PEGylated protein. Toxicol. Lett..

[74] Nganou-Makamdop K., Billingsley J.M., Yaffe Z., O’Connor G., Tharp G.K., Ransier A., Laboune F., Matus-Nicodemos R., Lerner A., Gharu L. (2018). Type I IFN signaling blockade by a PASylated antagonist during chronic SIV infection suppresses specific inflammatory pathways but does not alter T cell activation or virus replication. PLoS Pathog..

[75] Scopes R.K. (1994). Protein Purification.

